# Supplementary material for: Two-Parameter Quasi-Ballistic Transport Model for Nanoscale Transistors
Source: Sci Rep. 2019 Jan 24;9:525. doi: 10.1038/s41598-018-36692-7 (PMC6346087; doi:10.1038/s41598-018-36692-7)
Supplement: Supplementary file 1 — Appendix [file 41598_2018_36692_MOESM1_ESM.pdf]

Two-Parameter Quasi-Ballistic Transport Model for Nanoscale Transistors

Ji Ung Lee[1]*, Ramya Cuduvally[1], Prathamesh Dhakras[1], Phung Nguyen[1], and Harold L. Hughes[2]

[1]Colleges of Nanoscale Science and Engineering, SUNY-Polytechnic Institute, Albany, NY, 12203, USA. [2]US Naval Research Laboratory, Washington, DC, 20375, USA.
* Correspondence should be addressed to jlee1@sunypoly.edu

I. Appendix

Here, we show the origin of the two parameters, T and Δ, in our model. First, we illustrate using band diagrams the origin of two quasi-Fermi levels along the channel and relate them to the ballistic conductance. We show explicitly that these levels need to be defined locally, as opposed to taking their values from the source/drain contacts.

For a purely ballistic conductor with no scattering, Figure A1 (a) illustrates how the quasi-Fermi levels extend from the contacts to the channel (dotted lines). T, the transmission probability, is unity, but it can also be viewed as the fraction of the drain quasi-Fermi level that appears in the channel when the source is at ground. Here, the quasi-Fermi levels in the channel are those of the source/drain contacts. In other words, the source and drain quasi-Fermi levels from the contacts extend into the channel region.

With scattering, T <1. The band diagram that illustrates a uniform transmission along the channel is shown in Fig. A1(b). At the source end of the channel, T is also the fraction of the drain quasi-Fermi level that appears there.

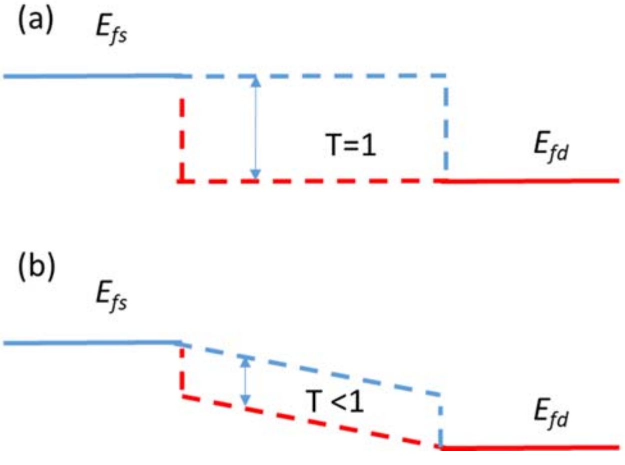

Fig. A1: Band diagram of (a) a purely ballistic conductor with T=1 and (b) a quasi-ballistic conductor with T<1. The region with dotted quasi-Fermi levels represents the channel region of the conductor. Here, T can be viewed as the fraction of E_{fd} that appears on the source end.

The band diagrams in Fig. A1 are consistent with the derivation of the conductance quantum for a single mode, as given in Equations A1 and A2. Eq. A1 can be derived from Eq. (1) in the low-temperature limit and by assuming a constant *T*.

$$I = \frac{2e}{h} T\left(E_{fs} - E_{fD}\right) \quad \text{(A1)}$$

$$G = I/V = \frac{2e^2}{h} T \quad \text{(A2)}$$

The equations above give the correct conductance quantum *G* as a function of *T*. A similar argument is provided in Ref. [1]. Fig. A1(b) transforms into Fig. 1A(a) if $T = 1$. In summary, two quasi-Fermi levels are needed to describe a quasi-ballistic conductor, and one can interpret *T* as the fraction of the drain quasi-Fermi level that appears at the source end.

The preceding point of view is different from the typical treatment of diffusive transport where only the local potential in the channel is needed to describe the current flow, as can be seen in the equation for the long-channel FET of Eq. (4). In fact, Fig. A1(a) would also apply to a purely ballistic MOSFET where the gate has perfect control of the channel potential. The same, however, cannot be said of Fig. A1(b) for a quasi-ballistic FET where the incomplete coupling of the gate to the channel creates a highly non-uniform potential profile along the channel. Instead, a modified band diagram is needed, resulting in Fig. 2 with two parameters to describe the transport as we discuss below.

In a MOSFET, the gate complicates the analysis, and one cannot use the band diagram for a conductor shown in Fig. A1(b) to represent a quasi-ballistic MOSFET. More importantly, *T* is no longer the fraction of E_{fd} that appears at the top of the source. If we assume that the gate "pegs" the band profile at the top of the source, *T* then no longer represents the difference E_{fs}-E_{fd} at the peak of the band diagram. Instead, in Fig. 2a, Δ replaces *T* of Fig. A1(b). Furthermore, Δ is expected to be independent of the channel length for a well-designed MOSFET. Our observation of a constant Δ in Fig. 6 supports this picture.

Thus, we have the origin of the two-parameter fit: T and Δ become decoupled in a quasi-ballistic MOSFET whereas they are the same in a quasi-ballistic conductor without a gate.

1 Datta, S. *Electronic Transport in Mesoscopic Systems*. (Cambridge University Press, 1995).
